# Inherited Metabolic Disorders: From Bench to Bedside

**DOI:** 10.3390/biomedicines12010174

**Published:** 2024-01-12

**Authors:** Tiago Fonseca, M. Fátima Macedo

**Affiliations:** 1 Faculdade de Medicina, Universidade de Coimbra, 3000-548 Coimbra, Portugal; tiagoofonsecaa@gmail.com; 2 CAGE, Instituto de Investigação e Inovação em Saúde (i3S), University of Porto, 4200-135 Porto, Portugal; 3 Departamento de Ciências Médicas, University of Aveiro, 3810-193 Aveiro, Portugal

Inherited metabolic disorders (IMDs), commonly referred to as inborn errors of metabolism, represent a spectrum of disorders with a defined (or presumed) primary genetic cause which disrupts the normal metabolism of essential molecules in the body [1]. From a biochemical perspective, these conditions include disorders of carbohydrate, amino acid, and fatty acid metabolism; lysosomal storage diseases (LSDs); organic acidemias; and mitochondrial disorders. Although these are relatively rare conditions, they represent a diverse array of disorders which encompass a significant amount of morbidity and mortality worldwide. A retrospective study conducted in the United Kingdom estimated the prevalence of inherited metabolic disorders to be 1 in 784 live births, with the most frequent diagnoses being mitochondrial disorders, followed by LSD and amino acid disorders [2].

This Special Issue aims to give an overview of various of such conditions, particularly focusing on LSDs like Mucopolysaccharidoses (MPS) and the Anderson–Fabry disease (AFD). It also delves into disorders related to the metabolism of carbohydrates and proteins, including Congenital Disorders of Glycosylation (CGD) and Glycogen Storage Disease (GSD) IV. All five papers submitted by international experts in the field were deemed suitable for publication following a rigorous peer-review process.

Amaral and colleagues conducted a comprehensive review of LSD, exploring the key cellular and molecular aspects and how various defects in lysosomal biology contribute to distinct diseases [3]. LSD comprise a group of inherited monogenic conditions marked by a progressive and multisystemic phenotype. Specific mutations in genes related to lysosomal proteins or non-lysosomal proteins crucial for lysosomal function can result in these diseases. Certain mutations lead to the accumulation of molecules such as sphingolipids, glycoproteins, and mucopolysaccharides within the lysosomes, leading to cellular damage. Consequently, a cascade of effects is generated, impacting cellular function through signaling abnormalities, defects in calcium homeostasis, oxidative stress, and inflammation. However, the mechanisms behind the pathogenesis of LSD is not yet fully understood [4]. LSDs inevitably affect multiple organs, showcasing heterogeneity even within the same disease. Onset can occur prenatally, early in life, or later in childhood or adulthood, with more severe manifestations observed in early-onset patients. With nearly 70 disorders identified, LSD can impact various body systems, with higher predilection for the central nervous, cardiovascular, skin, and skeletal systems. Beyond these “classic” organ involvements, additional systems, like the immune system, may also be affected.

The same group extended their exploration to elucidate how the accumulation of mucopolysaccharides in MPS impacts the immune system. MPS, a subset of LSD, is characterized by the intra-lysosomal buildup of undegraded or partially degraded glycosaminoglycans, which can be found in higher concentrations in urine, blood, and cerebrospinal fluid [5]. MPS II and VI, being X-linked and autosomal recessive diseases, respectively, lead to the intra-lysosomal accumulation of heparan sulfate and dermatan sulfate. Both MPS II and VI manifest a chronic and progressive course, featuring organomegaly, dysostosis multiplex, coarse facial features, and potential effects on hearing, vision, cardiovascular function, and joint mobility. Severe forms of MPS may result in recurrent upper respiratory infections, pneumonia, bronchitis, and middle ear infections, which can be partly attributed to impaired immune function. In their study, Lopes and colleagues described a significant decrease in the percentage of monocytes and NK cells in MPS VI patients compared to healthy individuals [6]. Additionally, there was an increase in the frequency of naïve T cells in MPS VI patients compared to healthy individuals. While there were no significant differences in the frequency of invariant natural killer T (iNKT) cells between MPS patients and healthy individuals, there was a shift in phenotype marked by a decrease in the frequency of CD161^+^ iNKT cells, a specific marker of NK cells, in MPS VI and MPS II patients compared with healthy individuals.

iNKT cells are immunomodulatory lipid-reactive lymphocytes which have been found to be decreased in frequency in several animal models of LSD, including AFD, Sandhoff disease, GM1 gangliosidosis, and Niemann–Pick C disease [7,8,9,10,11,12]. An elegant study by Espen and colleagues in acid sphingomyelinase deficiency (ASMD) illustrates a mechanism by which iNKT cells are decreased in LSD, and the study was extended to human patients. ASMD disease is characterized by the accumulation of sphingomyelin, a lipid that is able to prevent iNKT cell activation and, in this way, to reduce the numbers of iNKT cells when accumulated, in both animal models and patients [13].

Nevertheless, as mentioned earlier, the organic impact of LSD extends beyond the immune system. Specifically, one of the more frequently affected systems is the central nervous system. In the context of AFD, Zedde and colleagues brought attention to evidence supporting the connection between this lysosomal disease and Parkinson’s Disease (PD) by conducting a comprehensive literature review [14]. Lysosomal dysfunction, a well-established mechanism of PD pathogenesis, serves as a backdrop for interpreting the biological links between AFD and PD. The well-known model provided by the association between glucocerebrosidase (GBA) gene mutations and Gaucher disease is instrumental in the interpretation of the AFD findings. With GBA activity naturally decreasing with age, mutations in GBA can expedite this decline, reaching a critical threshold for substrate accumulation. This accumulation leads to the impairment of alpha-synuclein traffic, resulting in disruptions in the autophagy–lysosome system (ALS) and subsequent axonal degeneration [15]. These disruptions mirror features observed in the brains of α-galactosidase A (α-Gal A)-deficient mice, suggesting that insufficient α-Gal A activity in AFD may contribute to neurodegenerative processes akin to those seen in PD, namely the presence of aggregates of alpha-synuclein [16]. These findings align with similar observations in the human brains of PD patients and reinforce the association between AFD and PD [17]. Furthermore, studies measuring α-Gal A activity in human leukocytes and brain tissue found lower α-Gal A activity in PD patients compared to healthy controls, and patients with causal mutations of GLA exhibited a bradykinetic phenotype, a cardinal sign of PD, even in the absence of classical prodromal features [18,19].

The association between AFD and PD was initially signaled more than 20 years ago, when two AFD-diagnosed patients displayed clinical extrapyramidal phenotypes that were DOPA-responsive, later receiving a PD diagnosis [20,21].

In conclusion, the convergence of clinical evidence, lysosomal dysfunction mechanisms, and experimental findings, including bradykinetic phenotypes in individuals with causal GLA mutations, underscores a compelling association between AFD and PD. This linkage, reminiscent of the established relationship between GBA mutations and Gaucher disease, emphasizes shared biological pathways, such as α-Gal A deficiency and disruptions in the ALS, contributing to neurodegenerative processes observed in both AFD and PD.

In their case report, Kodríková and colleagues conducted a comprehensive glycoprofile analysis of a 9-year-old male patient harboring a novel missense variant in the SLC35A2 gene, presenting a mild phenotype of SLC35A2-linked CDG [22]. Despite manifesting delayed psychomotor development, hepatopathy, and short stature, the patient’s symptoms were relatively milder compared to previously reported cases. Remarkably, the study highlighted a distinct glycoprofile of transferrin in the patient, characterized by abnormal levels of hypogalactosylated N-glycans. Whole-exome sequencing identified a de novo mutation in the SLC35A2 gene, underscoring its involvement in observed glycosylation defects. The abnormal N-glycans identified hold promise as potential glycobiomarkers for SLC35A2-CDG, offering prospects for disease monitoring and treatment assessment, particularly in the context of galactose supplementation therapy. Case reports such as this, documenting previously unknown mutations in SLC35A2-CDG, play a crucial role in unraveling the complexities of rare metabolic diseases like CGD by enhancing insight into their biochemical pathways.

Wilke and colleagues presented a case series featuring five patients with GSD IV, each exhibiting different ages at presentation, cardiac phenotypes, and attempts at dietary intervention [23]. The study sheds light on the phenotypic spectrum of and therapeutic approaches to this rare disease, emphasizing the hepatic phenotype and the potential necessity for liver transplantation (LT) in progressive cases. The outcomes of LT in GSD IV patients are explored, with specific attention to cardiomyopathy, particularly dilated cardiomyopathy, and post-transplantation sepsis. The article underscores the variability in cardiac responses to LT and stresses the importance of periodic echocardiograms in GSD IV individuals. Genetic aspects of GSD IV are discussed, focusing on variants in the GBE1 gene and their associations with different phenotypes. The study also delves into the potential role of chitotriosidase as a biomarker for GSD IV, emphasizing the need for larger investigations. Additionally, a novel genetic variant, c.826G > C; p(Ala276Pro), associated with the GSD IV phenotype, is described for the first time. Overall, this case series contributes to a broader understanding of the diverse clinical spectrum of and therapeutic approaches to GSD IV, aiming to enhance treatment recommendations and diagnostic guidelines for this rare disorder.

In conclusion, this Special Issue thoroughly examines various rare metabolic diseases, offering valuable new insights. From exploring the molecular mechanisms and cellular pathways to unraveling novel and unknown genetic variants, as well as shedding light on new potential biomarkers, we hope for this Special Issue to cast a new light on the understanding of inherited metabolic disorders. By doing so, we aim to foster progress in diagnostics and treatment strategies and advance the overall knowledge of these disorders. We found the quality of the contributions to be commendable, and the Special Issue has gathered over 10,000 views as of today.
